# Editorial: Robotic perception, multi-robot exploration, mapping, and autonomous computing

**DOI:** 10.3389/frobt.2026.1974584

**Published:** 2026-09-03

**Authors:** Faiza Gul, Laith Abualigah, Rafal Szczepanski, Imran Mir

**Affiliations:** 1 Air University, Aerospace and Aviation, Kamra Kalan, Pakistan; 2 Al al-Bayt University, Mafraq, Jordan; 3 Institute of Engineering and Technology, Nicolaus Copernicus University in Torun, Torun, Poland

**Keywords:** autonomous computing, multi-robot exploration, path planning, robotic perception, robotic readiness, semantic mapping, SLAM

## 1 Introduction

Robotic perception, exploration, and mapping have progressed substantially in recent years, supported by advances in deep learning, sensor fusion, and probabilistic estimation. Robots are no longer designed exclusively for well-structured laboratory environments. They are increasingly expected to operate in settings that are dynamic, uncertain, and often resource-constrained, including agricultural facilities, offices, road networks, and healthcare infrastructures.

This shift brings several closely related challenges. Robots must be able to perceive and map environments that change over time, coordinate efficiently with other robots during exploration, interpret human instructions expressed in natural language, and operate within organizational and regulatory contexts that may either enable or limit their deployment. Addressing these Research Topic requires progress not only in algorithms and sensing technologies, but also in the broader systems in which autonomous robots are deployed.

This Research Topic was launched to bring together work on robotic perception, multi-robot exploration and coordination, mapping, and autonomous computing. The five published articles address visual SLAM in dynamic environments, language-grounded hierarchical path planning, hybrid multi-robot exploration, AI-defined autonomous vehicles, and the structural determinants of robotic readiness in healthcare systems. Although they approach autonomy from different perspectives, together they illustrate a continuum from low-level perception and planning to questions of adoption, governance, and societal integration.

## 2 Overview of the contributions

### 2.1 Perception and mapping in dynamic environments

Du et al. address a long-standing limitation of visual SLAM systems: their reduced accuracy in environments containing moving objects. Building on ORB-SLAM2, the authors propose GNV2-SLAM, which integrates a lightweight YOLOv8-based detector, GNV2, with a GhostNetV2 backbone, a CBAM attention module, and SCDown downsampling. The framework identifies and removes dynamic objects, such as cattle and workers, while point-line feature fusion is used to support more stable pose estimation.

The system was evaluated using the TUM benchmark and a physical inspection robot operating in a cowshed. GNV2-SLAM reduced the absolute trajectory error by more than 96% compared with ORB-SLAM2 while maintaining frame-processing times below 30 m. These results suggest that computationally efficient, dynamic-object-aware perception can make visual SLAM more suitable for cluttered and populated real-world environments.

### 2.2 Semantic mapping and hierarchical path planning

Taniguchi et al. consider robot navigation based on spoken instructions rather than explicit spatial coordinates. Their SpCoTMHP framework combines spatial concept formation with topometric semantic mapping in a unified probabilistic generative model. The approach supports approximate inference across semantic, topological, and metric representations of the environment.

In navigation experiments involving waypoint-based spoken commands, such as “go to the bedroom via the corridor,” SpCoTMHP improved both the weighted success rate of reaching the intended destination through the specified waypoint and the computation time when compared with a heuristic hierarchical baseline. The work demonstrates how concept-based hierarchical maps can support navigation that is both interpretable to users and directly responsive to natural-language instructions.

### 2.3 Multi-robot exploration

Gul et al. propose EMREL, a hybrid strategy for multi-robot exploration in unknown environments. The method combines the deterministic Coordinated Multi-Robot Exploration formulation, which uses a shared Frontier Information Structure to prioritize neighboring cells, with the stochastic Crayfish Optimizer, which refines the search over a wider area.

The authors evaluate EMREL on both standard and complex maps and compare it with contemporary exploration algorithms. The reported results indicate improvements in exploration rate and temporal efficiency, together with a lower number of failed runs. The study therefore points to the potential value of combining structured frontier-based exploration with metaheuristic search mechanisms in multi-robot systems.

### 2.4 Autonomy beyond the laboratory: AI-defined vehicles

De Vincenzi et al. extend the discussion of robotic autonomy to road vehicles equipped with adaptive and learning-based capabilities. Their review introduces AI-defined vehicles (AIDVs) as a distinct class of systems in which interaction, adaptability, sustainability, and ethical governance are treated as central design considerations rather than secondary features.

The article offers an initial conceptualization of AIDVs, distinguishes them from existing vehicle categories, and discusses risks associated with adaptive artificial intelligence. It also proposes a preliminary roadmap for integrating such vehicles into Intelligent Transportation Systems. In this way, the contribution connects robotic autonomy and multi-agent coordination with the wider technological and societal infrastructure of future transportation systems.

### 2.5 Systemic readiness for robotic deployment

Mukherjee and Ali shift attention from robotic algorithms to the conditions required for their large-scale adoption. Their study examines how prepared healthcare systems are to integrate robotics by using 31 digital-health maturity indicators across 169 countries. The authors derive latent readiness dimensions related to perception and interoperability, governance and coordination, and workforce and regulatory reliability.

Using unsupervised clustering and supervised machine-learning methods, including random forest and XGBoost models with reported accuracies of up to 0.98, the study identifies distinct national maturity regimes. These regimes are validated against World Bank and World Health Organization classifications. The SHAP analysis further identifies governance and coordination readiness as the strongest predictor of cluster membership. The findings underline that technological capability alone is insufficient; coherent governance, regulation, and institutional capacity are also necessary for the responsible deployment of autonomous robotic systems.

## 3 Discussion and future directions

Despite addressing different application domains—livestock inspection, indoor service navigation, exploration of unknown environments, road transportation, and global health policy—the five contributions share several important themes. First, robotic perception must remain reliable in environments shaped by motion, uncertainty, and incomplete information. Second, exploration and planning can benefit from combining structured, deterministic methods with adaptive or stochastic optimization. Third, semantic representations are essential for making autonomous behavior more understandable and usable for people. Finally, the successful deployment of robotics depends not only on technical performance, but also on institutional, regulatory, and organizational readiness.

Several open research challenges remain. These include decentralized multi-robot coordination under limited or unreliable communication, semantic scene understanding that generalizes across environments and application domains, and the translation of readiness assessments into practical policies for robotic deployment. Further work will also be needed to connect advances in perception, planning, and learning with the safety, governance, and human-centered requirements of real-world autonomous systems.

We hope that this Research Topic will encourage further original research and review articles that advance robotic perception, exploration, mapping, and autonomous computing. We sincerely thank all contributing authors and reviewers for their time, expertise, and valuable contributions to this Research Topic.

